# Occurrence of Potential Prescribing Cascades After Hospital Discharge: A Cohort Study

**DOI:** 10.1002/pds.70305

**Published:** 2026-01-05

**Authors:** Atiya K. Mohammad, Johanna H. M. Driessen, Jacqueline G. Hugtenburg, Alex Marmorale, Carl Siegert, Patricia M. L. A. van den Bemt, Petra Denig, Fatma Karapinar‐Çarkıt

**Affiliations:** ^1^ Department of Clinical Pharmacy OLVG Hospital Amsterdam the Netherlands; ^2^ Department of Clinical Pharmacy and Pharmacology University Medical Centre Groningen Groningen the Netherlands; ^3^ Department of Clinical Pharmacy & Toxicology Maastricht University Medical Center+ Maastricht the Netherlands; ^4^ Department of Clinical Pharmacy, CARIM, Cardiovascular Research Institute Maastricht Maastricht University Maastricht the Netherlands; ^5^ Department of Clinical Pharmacology and Pharmacy Amsterdam UMC, VUMC Amsterdam the Netherlands; ^6^ Epic Systems Corporation Verona Wisconsin USA; ^7^ Department of Internal Medicine OLVG Hospital Amsterdam the Netherlands

**Keywords:** continuity of care, hospital discharge, inappropriate prescribing, prescribing cascades, transitions in care

## Abstract

**Purpose:**

A prescribing cascade (PC) occurs when a medication (index) causes an adverse drug reaction (ADR), which is addressed by prescribing additional medication (marker). Medication initiated in the hospital may cause post‐discharge ADRs and PCs, especially when multiple healthcare providers are involved. The study aimed to assess the cumulative incidence of potential PCs post‐discharge and identify the healthcare providers involved in prescribing the marker medication.

**Methods:**

A cohort study was conducted among adult patients admitted in one hospital between 2019 and 2023, who initiated medication associated with preselected PCs (*n* = 20). A PC was defined as the initiation of a marker medication which may be intended to treat an ADR induced by the index medication. Data from the hospital and the Nationwide Medication Record System were used to identify potential PCs post‐discharge. The primary outcome was the cumulative incidence of PCs, estimated for PCs with ≥ 10 patients initiating the index medication. The secondary outcome was the percentage of cases where the marker medication was prescribed by a healthcare provider outside the hospital, for PCs with ≥ 10 patients initiating the marker medication. Descriptive statistics were used.

**Results:**

Among 24 282 patients initiating index medication, 502 potential PCs were observed. The cumulative incidence was estimated for 17 PCs, ranging from 0% to 12.3%. Across 12 PCs with ≥ 10 patients, percentages of marker medications prescribed outside the hospital ranged from 31.8% to 92.8%.

**Conclusion:**

The cumulative incidence of potential PCs post‐discharge can be substantial with marker medication often initiated by healthcare providers outside the hospital.

## Key Points

- The cumulative incidence of potential prescribing cascades within 1 year after hospital discharge ranged from 0% to 12.3%, including four with previous strong evidence and current incidences of at least 4%.
- The medication used to treat a potential adverse drug reaction indicative for a prescribing cascade after discharge was often prescribed by a healthcare provider outside the hospital.
- These findings highlight the risks associated with transitions of care and indicate room for improvement of post‐discharge medication management.

## 1 Introduction

Medication‐related harm, including adverse drug reactions (ADRs) appears to be common after transitions of care, such as at hospital discharge [1]. When medication changes are implemented during a hospital stay, ADRs may manifest after hospital discharge [2]. It is therefore essential that these potential ADRs are monitored and managed effectively after discharge. With insufficient information exchange to the patient and next healthcare providers, there is an increased risk that a healthcare provider fails to recognize an ADR and treats this as a new medical condition with a new prescription. This is known as a prescribing cascade [3, 4].

Previous observational studies have identified the occurrence of potential prescribing cascades and some have explored their incidence [5, 6, 7]. For example, it was observed that between 1.4% and 4.9% of patients initiating a calcium channel blocker (CCB) were subsequently treated with diuretics potentially for peripheral edema caused by the CCB [8, 9, 10, 11]. In one study, the rate was compared to controls, for whom the incidence of diuretics initiation was 0.5%–0.7% [10]. Another study among primary care patients found that in 64% of patients, the diuretic in this potential prescribing cascade was prescribed by a healthcare provider different from the one who prescribed the CCB [12].

Most research on potential prescribing cascades has been conducted within a single setting [5, 7], creating a knowledge gap regarding those occurring after transitions in care and the involvement of different healthcare providers. Therefore, the aim of this study was to assess the occurrence of potential prescribing cascades after hospital discharge. The secondary aim was to identify whether the additional medication was started by a healthcare provider from the hospital where the medication was initiated or by a healthcare provider from another setting.

## 2 Methods

### 2.1 Study Design

A cohort study was conducted among adult patients, who had initiated new medication involved in 20 potential prescribing cascades in a teaching hospital (OLVG Amsterdam, the Netherlands) between January 2019 and December 2023.

### 2.2 Study Setting

In the teaching hospital, medication orders issued during hospitalization and at outpatient hospital visits are recorded in the hospital's Electronic Health Record (EHR) system. Medication dispensed before or after hospitalization can be retrieved from the Nationwide Medication Record System (NMRS) [13]. The NMRS includes all medications dispensed by community pharmacies for prescriptions issued by healthcare providers, such as those from other hospitals or general practitioners. Over‐the‐counter (OTC) medication is only included in the NMRS when it is documented by the community pharmacy. Typically, OTC medications are documented when a patient purchases them at the pharmacy or during consultations, when all relevant medications in use are recorded. However, not all community pharmacies routinely document OTC medications. In routine practice, the NMRS is queried automatically at each hospital contact (i.e., admission, outpatient clinic visit, telephone follow‐up) to generate a longitudinal medication overview. For the current study, we used these routinely collected data to capture patients' medication use. For the current study, we used these routinely collected data to capture patients' medication use. Patients must provide consent to exchange medication information through the NMRS.

### 2.3 Ethics

This study was approved by the local ethics committee (ACWO‐MEC; OLVG, Amsterdam; ID number: 22169). Dutch legislation does not require informed consent for studies processing routinely collected anonymous data that do not affect the patient's integrity [14].

### 2.4 Selection

In a previous study, an expert team of both physicians and pharmacists assessed which potential prescribing cascades were problematic, where this was defined as follows: *the benefits of a prescribing cascade do not outweigh the risks for the health of a patient* [10]. From this previous study, 20 potentially problematic prescribing cascades were selected for which a statistically significant positive association was found using Dutch dispensing data between the medication potentially causing the ADR (index medication) and the medication used to treat the ADR (marker medication) [15]. Examples of selected prescribing cascades include the use of ACE‐inhibitors potentially causing cough treated with cough suppressants and dihydropyridines potentially causing edema treated with diuretics. The cutoff used to select prescribing cascades in the current study was having an adjusted sequence ratio (aSR) ≥ 1.5 as quantified with prescription sequence symmetry analysis in this previous study [15]. To classify the likelihood that these selected prescribing cascades occur in clinical practice, we distinguished potential prescribing cascades having shown weak associations (aSRs between 1.5 and 2.5), moderate associations (aSR between 2.5 and 3.5), and strong associations (aSR larger than 3.5) in this study conducted in the Netherlands [15]. In addition, we used a recent review study to classify the level of evidence for the prescribing cascades in general [7]. In this review Shahid et al. classified the evidence for potential prescribing cascades as being poor, fair, moderate or strong.

### 2.5 Study Population

All adult patients (≥ 18 years) admitted to any department of the teaching hospital between 2019 and 2023 were included in the study if they initiated an index medication (i.e., incident users) during their hospital stay, which could potentially lead to one of the selected 20 potential prescribing cascades. Services from which patients were included varied and represented a broad range of medical specialties to enhance the generalizability of the findings. Incident use was defined as not having a dispensing or medication order for the index medication in the previous 12 months, that is, the lookback period [11]. Since the lookback period in the NMRS is limited to 8 months, medication orders within the hospital were used to extend the lookback period to a full 12 months, ensuring accurate identification of incident use.

After hospital discharge, patients with less than 1 year of follow‐up data in the NMRS (e.g., because there was no follow‐up visit or contact to the hospital, or loss to follow‐up, for example, due to death) were excluded. Also excluded were patients for whom the time between the hospital discharge order and actual dispensing of the index medication post‐discharge exceeded 90 days, indicating insufficient follow‐up on outpatient dispensing [13]. While medication is typically supplied for a 3‐month period post‐discharge, this may vary by patient needs and prescribed preferences, and no adjustment was made for tailored quantities at hospital discharge in this study. To ensure that the marker medication was prescribed in response to a potential ADR rather than for an unrelated condition, patients were excluded if they had been dispensed the marker medication in the year before initiating the index medication suggesting non‐incident use of the marker medication. Additionally, a 7‐day blackout period was applied between the start dates of the index and marker medications, as most ADRs are not treated this soon after the start of an index medication [11, 12].

To define continuous use of the index medication at the time the marker medication was dispensed, a maximum gap of 7 months was allowed between the dispensing date of the first marker medication and the dispensing date of the previous index medication in the NMRS. Medication is typically dispensed for 3 months in the Netherlands. An additional stock period of 4 months was included to account for medication the patient may still be using due to overlapping dispensings or using less medication than initially prescribed, aligning with previous studies [11, 16]. This could be due to, for example, repeat dispensings soon after each other or dose reductions that are not always documented.

Finally, patients for whom the marker medication was started after the 12 months follow‐up period (i.e., the exposure window) were excluded [17].

The occurrence of a potential prescribing cascade was defined as the incident use of the index medication at hospital discharge, its continued use post‐discharge followed by the incident dispensing of a marker medication within 12 months.

### 2.6 Outcomes

The primary outcome was the cumulative incidence per potential prescribing cascade after hospital discharge. This was estimated by dividing patients experiencing a potential prescribing cascade by those who initiated new index medication at discharge. This was calculated for prescribing cascades with ≥ 10 patients initiating the index medication, to ensure a minimal sample size.

The secondary outcome was the percentage of prescribing cascades for which the marker medication was prescribed by healthcare providers outside of the teaching hospital. This measure was chosen to reflect the typical extent to which the responsibility for continuing or initiating the marker medication shifted to providers in other settings, thereby highlighting the role of transitions of care. This was calculated for prescribing cascades with ≥ 10 patients initiating the marker medication, to ensure a minimal sample size.

### 2.7 Data‐Collection

Patient data extracted from the teaching hospital's EHR system included a patient identifier, year of birth, sex, length of hospital stay, department of admission, index and/or marker medication order including date of the discharge order and the Anatomical Therapeutic Chemical (ATC) code of the medication. Dispensing data extracted from the NMRS included the index and/or marker medication order and the dispensing date. All data was queried with Microsoft SQL Server Management Studio (Microsoft Corporation, Redmond, WA, USA) and collected in MS Excel 2016 (Microsoft Corporation, Redmond, WA, USA).

### 2.8 Analysis

Data‐analysis was performed using descriptive statistics. All analyses were performed in IBM SPSS version 29 (IBM Corporation, New York, USA).

## 3 Results

**FIGURE 1:**
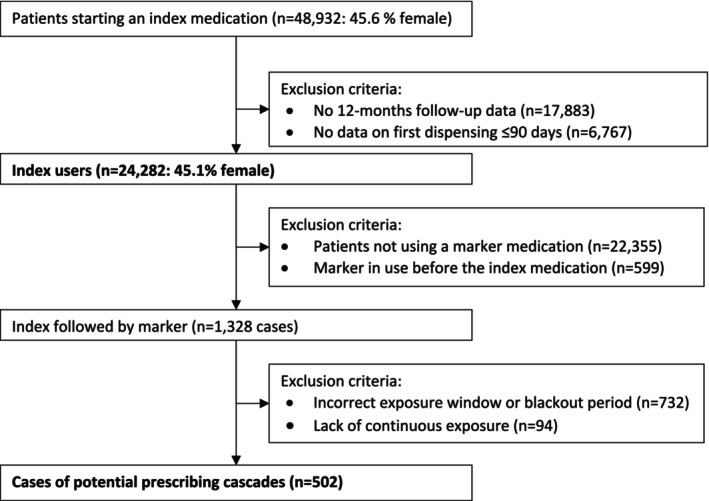
The flow of cases within the study period for 20 potential prescribing cascades.

For the 20 selected potential prescribing cascades, 48 932 patients started an index medication in the hospital (Figure 1). Of these patients, 17 883 were excluded because they lacked 12‐months follow‐up data and 6767 were excluded because no first dispensing data was found within 90 days. Of the remaining 24 282 incident index users, 1328 patients received the related marker medication after the index medication. Of these, 502 cases complied with the parameters of a potential prescribing cascade (Figure 1). For these potential prescribing cascades, 77% (*n* = 386) were observed in unique individuals. Three prescribing cascades were excluded from further analysis because fewer than 10 patients initiated an index medication (i.e., lithium potentially causing hypothyroidism treated with thyroid hormones, lithium potentially causing parkinsonism treated with dopaminergics, and lithium potentially causing tremor treated with propranolol, see Appendix 1, Supporting Information). For the remaining 17 potential prescribing cascades, the mean age of patients experiencing a potential prescribing cascades was 68.9 years (standard deviation 11.8). The cumulative incidence ranged between 0% and 1.0% for nine potential prescribing cascades and exceeded 1.0% for eight potential prescribing cascades (Table 1). The highest cumulative incidence (12.3%) was found for the potential prescribing cascade of angiotensin‐converting enzyme (ACE) inhibitor potentially causing urinary tract infections treated with antibacterials. This is a prescribing cascade that was classified as having shown a weak significant association in a previous Dutch database study and strong evidence from a systematic review (Table 1). For other prescribing cascades with cumulative incidences of more than 2%, the evidence when assessed in the systematic review had also been classified as being strong with associations in the Dutch study varying from weak to strong (Table 1).

**TABLE 1:** Prescribing cascades observed with their general likelihood and occurrences in the current study.

Prescribing cascade								
Index medication—potential adverse drug reaction—marker medication	Likelihood based on aSR [15], evidence synthesis [7]^a^	Number starting index	Number starting marker	Cumulative incidence (%)	Mean age [SD]^b^	Female (%)^b^	Start marker by HCP outside of the hospital	Most common department of admission^b^
ACE‐inhibitors—cough—antibacterials for cough (systemic use)	Weak aSR Evidence not assessed	1286	51	4.0	69.3 [15]	35.3	32 (62.7%)	Cardiology
ACE‐inhibitors—cough—antihistamines (systemic use)	Weak aSR Evidence not assessed	1267	37	2.9	67.7 [16]	54.1	31 (83.8%)	Cardiology
ACE‐inhibitor—cough—antitussives	Moderate aSR Strong evidence	1268	69	5.4	71.5 [12]	44.9	64 (92.8%)	Cardiology
ACE‐inhibitors—erectile dysfunction—Medications used in erectile dysfunction	Weak aSR Moderate evidence	1411	22	1.6	65.4 [15]	4.5	17 (77.3%)	Cardiology
ACE‐inhibitors—urinary tract infections—antibacterials for urinary tract infections (systemic use)	Weak aSR Strong evidence	1037	128	12.3	70.6 [14]	38.3	92 (71.9%)	Cardiology
Amiodarone—hypothyroidism—thyroid hormones	Strong aSR Strong evidence	428	17	4.0	79.4 [10]	35.3	8 (47.1%)	Cardiology
Angiotensin II receptor blockers—erectile dysfunction—medications used in erectile dysfunction	Weak aSR Evidence not assessed	1209	10	0.8	67.7 [12]	0	5 (50.0%)	Cardiology
Antipsychotics—hyperprolactinemia or oligomenorrhea—prolactin inhibitors	Weak aSR Moderate evidence	177	0	—	—	—	—	—
Antipsychotics—Parkinsonism—tertiary amines/dopaminergics	Weak aSR Evidence not assessed	169	1	0.6	—	—	—	—
Beta blocking agents—erectile dysfunction—medications used in erectile dysfunction	weak aSR evidence not assessed	2881	17	0.6	63.9 [15]	5.9	13 (76.5%)	Cardiology
Dihydropyridines—edema peripheral—high‐ceiling diuretics	Weak aSR Strong evidence	1288	81	6.3	70.9 [14]	44.4	36 (44.4%)	Cardiology
Dihydropyridines—Erectile dysfunction—medications used in erectile dysfunction	Weak aSR Moderate evidence	1395	12	0.9	67.3 [10]	0	9 (75.0%)	Cardiology
HMG CoA reductase inhibitors—cognitive impairment—anti‐dementia medications	Moderate aSR Evidence not assessed	2786	3	0.1	82.3 [6]	33.3	—	—
HMG CoA reductase inhibitors—erectile dysfunction—medications used in erectile dysfunction	Weak aSR Moderate evidence	2742	27	1.0	66.3 [11]	3.7	21 (77.8%)	Cardiology
Low‐ceiling diuretics—erectile dysfunction—medications used in erectile dysfunction	Weak aSR Moderate evidence	302	5	1.7	60.4 [10]	—	—	—
Non‐dihydropyridines—erectile dysfunction—medications used in erectile dysfunction	Weak aSR Moderate evidence	671	0	—	—	—	—	—
Proton pump inhibitors— *Clostridium difficile* infection—intestinal antiinfectives	Moderate aSR Moderate evidence	3945	22	0.6	62.6 [16]	45.5	7 (31.8%)	Internal medicine

Abbreviations: ACE = angiotensin‐converting enzyme, aSR = adjusted sequence ratio, HCP = healthcare provider, HMG CoA = 3‐hydroxy‐3‐methyl‐glutaryl‐coenzyme A, SD = standard deviation.

^a^ Strong evidence: At least two high‐quality observational studies show a significant positive association between index and marker drug use, with no high‐quality study showing a significant inverse association. Moderate evidence: One high‐quality observational study shows a significant positive association, with no high‐quality study showing a significant inverse association.

^b^ Calculated over the number of patients starting their marker medication.

For 12 out of the 17 potential prescribing cascades, at least 10 patients initiated the marker medication. Across these prescribing cascades, the percentage with marker medication prescribed outside the hospital ranged from 31.8% to 92.8%. The highest percentage (> 90%) was found for the prescribing cascade of ACE‐inhibitor causing cough treated with antitussives.

## 4 Discussion

Cumulative incidences of 17 potential prescribing cascades within 12 months after hospital discharge ranged from zero to 12.3%. Across 12 prescribing cascades with at least 10 patients, percentages of marker medications initiated by healthcare providers from outside the hospital ranged from 31.8% to 92.8%.

This is a first study assessing the occurrence of potential prescribing cascades after hospital discharge, which limits the direct comparison of our findings with previous studies that have mostly been conducted in primary care without focusing on transitions in care [7, 15]. Our focus was on potential prescribing cascades that were previously assessed as problematic and regarded clinically relevant by experts [15]. The most common potential prescribing cascades in our study have previously shown strong supporting evidence [7]. The five most common ones were among the top‐15 in the previous study, showing weak to strong associations in primary care [15]. Such variation is to be expected given the differences in study methods and populations. Fewer females appeared to be exposed to potential prescribing cascades in our study. This is surprising, because women usually report more ADRs than men [18]. This may suggest that healthcare providers are more aware of ADRs in women, leading to less prescribing cascades, but this was not observed in the previous study [15]. This is more likely due to the fact that there were less females in our current study population. Some cascades are more plausible given the Dutch context; for instance, codeine is rarely prescribed for pain in the Netherlands, increasing the likelihood that such prescriptions after initiation of an ACE‐inhibitor were intended to treat ACE‐inhibitor induced cough.

We used a 12‐month exposure window for the marker medication to be initiated as is used most often in studies that evaluate prescribing cascades [15, 17]. However, certain ADRs may occur outside this timeframe. This could explain why prescribing cascades related to well‐known ADRs, such as tremor or hypothyroidism induced by lithium, were not observed in this study [19]. These ADRs may manifest later and the number of patients initiating these medications was small in this dataset.

The present study confirms the findings of previous studies showing that multiple healthcare providers are frequently involved in potential prescribing cascades [12, 20]. One study found that multiple HCPs were involved in almost two‐thirds of patients who were prescribed diuretics after initiating a calcium‐channel‐blocker [12]. Another study found that multiple healthcare providers were involved in nearly half of the patients who were prescribed anticholinergic medication after initiating dementia treatment [20].

### 4.1 Implications for Practice

Our study's observation of medication initiation post‐discharge—which may be intended to treat an ADR from medication started during hospitalization—highlights the need for adequate follow‐up care. Studies show that information regarding discharge medication may be incomplete [21], so effective communication between healthcare providers is crucial to ensure continuity of care and prevent unnecessary medication initiation. Many of the potential prescribing cascades found in this study involved well‐known ADRs, such as cough induced by ACE‐inhibitor use or edema induced by dihydropyridines. Healthcare providers should monitor ADRs and review medication regimens after discharge to identify and manage ADRs, particularly for older adults as the mean age in this study was generally over 65 years for 11 potential prescribing cascades.

### 4.2 Strengths and Limitations

A strength of the present study is its focus on clinically relevant prescribing cascades, achieved by combining medication data from a hospital EHR system and the NMRS. It should be noted, however, that the assessed cumulative incidence rates reflect potential prescribing cascades. The rates of actual prescribing cascade are expected to be lower, as marker medications can be prescribed for different symptoms. On the other hand, the incidence rates for potential prescribing cascades involving antitussives and antihistamines may be underestimated, as the use of OTC medications is often not accounted for. Another limitation is the exclusion of a substantial number of patients with insufficient follow‐up data, either because they did not consent to NMRS queries or did not have a follow‐up contact or visit to the hospital. This may have introduced selection bias affecting the cumulative incidence rates. This group likely includes a heterogeneous mix of patients, such as those who did not consent to NMRS data exchange, patients in long‐term care facilities, and those whose care was primarily transferred to primary care providers. As the characteristics of these patients are unknown, it is not possible to determine whether their exclusion led to an underestimation or overestimation of the observed incidence rates.

Although the study identified potential prescribing cascades that were selected based on the strength of the association in a previous study, we may have missed other frequently hospital‐initiated medications that were not included in our initial study—like the commonly observed antidepressants and Parkinson cascade—due to our study's focus on a limited set of medications. Additionally, the inclusion of only one hospital limited the ability to study prescribing cascades with small numbers of incident users. Expanding future research to include more hospitals and a broader range of prescribing cascades could improve the representativeness and generalizability of these findings to routine practice.

## 5 Conclusion

The cumulative incidences of potential prescribing cascades observed after hospital discharge indicate that there is room for improvement in managing ADRs to reduce prescribing cascades. In many cases, the medication which may have been added to treat a potential ADR was prescribed by healthcare providers from outside the hospital where the initial medication was started.

### 5.1 Plain Language Summary

When patients start a new medication in the hospital, it sometimes causes side effects that present themselves after hospital discharge. Additional medications can be prescribed to treat these side effects. This is known as a prescribing cascade. Prescribing cascades can result in unnecessary medication use and potential harm. This study investigated how often prescribing cascades occur after hospital discharge and which healthcare providers are initiating the additional medications. Data between 2019 and 2023 from one hospital were analyzed, focusing on patients who started specific medications, which may lead to prescribing cascades. These patients were followed for 1 year to assess whether they received another medication to treat a possible side effect. Among more than 24 000 patients, 502 cases of potential prescribing cascades were observed. The likelihood of this happening varied depending on the medication, ranging from 0% to 12.3%. Notably, the additional medications were often prescribed by healthcare providers outside the hospital. This suggests that prescribing cascades frequently extend beyond hospital care, highlighting the need for better communication and coordination between hospital and community healthcare providers.

## Author Contributions

Study concept and design: F.K.‐Ç., P.D., P.M.L.A.B., J.G.H., C.S. Acquisition of data: F.K.‐Ç., A.K.M., A.M. Data analysis: F.K.‐Ç., A.K.M., A.M., J.H.M.D. Interpretation of data: F.K.‐Ç., A.K.M., P.D., P.M.L.A.B., J.G.H., J.H.M.D. Preparation of manuscript: A.K.M., F.K.‐Ç., P.D., P.M.L.A.B., J.G.H. Critical feedback on manuscript: All authors.

## Funding

The OLVG hospital has supported the study with a non‐conditional grant (Grant Number: 595507). The funder had no role in the study design, data collection, data analysis, decision to publish, or preparation of the manuscript.

## Ethics Statement

This study was approved by the local ethics committee, Adviescommissie Wetenschappelijk Onderzoek‐Medisch‐Ethische Commissie (ACWO‐MEC; OLVG Hospital, Amsterdam [ID number: 22169]).

## Conflicts of Interest

The authors declare no conflicts of interest.

## Supporting information


**Appendix 1.** Demographics of the index users for each of the 20 selected prescribing cascades.

## Data Availability

The dataset generated during and/or analyzed during the current study is available from the corresponding author on reasonable request.
