# Supplementary material for: Occurrence of Potential Prescribing Cascades After Hospital Discharge: A Cohort Study
Source: Pharmacoepidemiol Drug Saf. 2026 Jan 5;35(1):e70305. doi: 10.1002/pds.70305 (PMC12768529; doi:10.1002/pds.70305)
Supplement: Supplementary file 1 — Appendix 1. Demographics of the index users for each of the 20 selected prescribing cascades. [file PDS-35-e70305-s001.docx]

**Supplementary materials**

**Title: Occurrence of potential prescribing cascades after hospital discharge: a cohort study**

**Running head: Prescribing cascades after hospital discharge**

**Appendix 1 – Demographics of the index users for each of the 20 selected prescribing cascades**

|  | Prescribing cascade | | |  |  |  |
| --- | --- | --- | --- | --- | --- | --- |
|  | *Index medication (ATC-code)* | *Adverse drug reaction* | *Marker medication (ATC-code)* | *Number starting index* | *Mean age [sd]* | *Female (%)* |
| *1* | ACE-inhibitors (C09A, C09B) | Cough | Antibacterials used for cough (systemic use) (J01A, J01B, J01F, J01G, J01R) | 1,286 | 68.3 [14] | 41.8 |
| *2* | ACE-inhibitors (C09A, C09B) | Cough | Antihistamines (systemic use) (R06A) | 1,267 | 68.7 [14] | 39.9 |
| *3* | ACE-inhibitor (C09A, C09B) | Cough | Antitussives (R05) | 1,268 | 68.2 [17] | 40.0 |
| *4* | ACE-inhibitors (C09A, C09B) | Erectile dysfunction | Medications used in erectile dysfunction (G04BE) | 1,411 | 68.7 [14] | 43.2 |
| *5* | ACE-inhibitors (C09A, C09B) | Urinary tract infections | Antibacterials used for urinary tract infections (systemic use) (J01C, J01D, J01E, J01M, J01X) | 1,037 | 67.6 [14] | 38.2 |
| *6* | Amiodarone (C01BD01) | Hypothyroidism | Thyroid hormones (H03AA) | 428 | 74.4 [10] | 36.7 |
| *7* | Angiotensin II receptor blockers (C09C, C09D) | Erectile dysfunction | Medications used in erectile dysfunction (G04BE) | 1,209 | 69.3 [14] | 41.8 |
| *8* | Antipsychotics (N05A) | Hyperprolactinemia or Oligomenorrhea | Prolactin inhibitors (G02CB) | 177 | 69.0 [17] | 48.6 |
| *9* | Antipsychotics (N05A) | Parkinsonism | Tertiary amines/ Dopaminergics (N04AA, N04B) | 169 | 68.4 [17] | 47.3 |
| *10* | Beta blocking agents (C07) | Erectile dysfunction | Medications used in erectile dysfunction (G04BE) | 2,881 | 67.8 [14] | 46.2 |
| *11* | Dihydropyridines (C08C, C08E, C08G, C09DB, C09BB) | Edema peripheral | High-ceiling diuretics (C03C) | 1,288 | 68.7 [14] | 45.5 |
| *12* | Dihydropyridines (C08C, C08E, C08G, C09DB, C09BB) | Erectile dysfunction | Medications used in erectile dysfunction (G04BE) | 1,395 | 69.3 [14] | 48.0 |
| *13* | HMG CoA reductase inhibitors (C10AA, C10B) | Cognitive impairment | Anti-dementia medications (N06D) | 2,786 | 69.0 [12] | 42.7 |
| *14* | HMG CoA reductase inhibitors (C10AA, C10B) | Erectile dysfunction | Medications used in erectile dysfunction (G04BE) | 2,742 | 69.0 [12] | 43.6 |
| *15* | Lithium (N05AN) | Hypothyroidism | Thyroid hormones (H03AA) | 7 | 56.1 [13] | 42.9 |
| *16* | Lithium (N05AN) | Parkinsonism | Tertiary amines/ Dopaminergics (N04AA, N04B) | 7 | 56.1 [13] | 42.9 |
| *17* | Lithium (N05AN) | Tremor | Propranolol (C07AA05) | 6 | 55.2 [28] | 50.0 |
| *18* | Low-ceiling diuretics (C03A, C03B) | Erectile dysfunction | Medications used in erectile dysfunction (G04BE) | 302 | 67.6 [14] | 43.7 |
| *19* | Non-dihydropyridines (C08D) | Erectile dysfunction | Medications used in erectile dysfunction (G04BE) | 671 | 68.6 [12] | 63.6 |
| *20* | Proton pump inhibitors (A02BC) | Clostridium difficile infection | Intestinal antiinfectives (A07A) | 3,945 | 60.3 [17] | 51.5 |

ATC: anatomical therapeutic classification, sd: standard deviation, ACE: angiotensin converting enzyme, HMG CoA: 3-hydroxy-3-methyl-glutaryl-coenzyme A.
